# Expression profile of circular RNAs in infantile hemangioma detected by RNA-Seq

**DOI:** 10.1097/MD.0000000000010882

**Published:** 2018-05-25

**Authors:** Jun Li, Qian Li, Ling Chen, Yanli Gao, Jingyun Li

**Affiliations:** Department of Plastic and Cosmetic Surgery, Maternal and Child Health Medical Institute, The Affiliated Obstetrics and Gynecology Hospital of Nanjing Medical University, Nanjing Maternity and Child Health Care Hospital, Nanjing, China.

**Keywords:** biomarker, circular RNA, infantile hemangioma, micro-RNA

## Abstract

**Background:**

Circular RNAs (circRNAs) have emerged as a novel class of widespread non-coding RNAs, and they play crucial roles in various biological processes. However, the characterization and function of circRNAs in infantile hemangioma (IH) remain elusive.

**Methods:**

In this study, we used RNA-Seq and circRNA prediction to study and characterize the circRNAs in IH tissue and a matched normal skin control. Specific circRNAs were verified using real-time polymerase chain reaction.

**Results and Conclusion:**

We found that of the 9811 identified circRNAs, 249 candidates were differentially expressed, including 124 upregulated and 125 downregulated circRNAs in the IH group compared with the matched normal skin control group. A set of differentially expressed circRNAs (in particular, hsa_circRNA001885 and hsa_circRNA006612 expression) were confirmed using qRT-PCR. Gene ontology and pathway analysis revealed that compared to matched normal skin tissues, many processes that were over-represented in IH group were related to the binding, protein binding, gap junction, and focal adhesion. Specific circRNAs were associated with several micro-RNAs (miRNAs) predicted using miRanda. Altogether, our findings highlight the potential importance of circRNAs in the biology of IH and its response to treatment.

## 1 Introduction

Infantile hemangioma (IH) is the most common vascular tumor in infants.^[1]^ It is characterized by an initial proliferation during infancy, followed by spontaneous involution over the next 5 to 10 years.^[2]^ Some IHs show such a severe progression that they lead to tissue and organ damage and in some cases become life-threatening.^[3]^ The cause of IH is reported to be highly associated with fetal hypoxic stress.^[4]^ However, the exact atiopathogeny underlying IH is still to be fully understood.^[4]^ It is therefore necessary to deeply explore the regulatory processes for a better understanding IH pathogenesis.

Circular RNAs (circRNAs) comprise a novel class of widespread non-coding RNAs that are predominantly generated by back-splicing of exons in eukaryotic genomes.^[5]^ Acting as microRNA sponges, circRNAs regulate multiple biological processes such as cancer, heart development, and liver diseases.^[6–8]^ CircRNAs have been reported to be abundant, conserved and stable in the cytoplasm. Specific circRNAs are correlated with hypoxia-regulated vascular cells,^[9]^ indicating that circRNAs may be involved in angiogenesis. The circRNA_000203 enhances the expression of fibrosis-associated genes by derepressing targets of miR-26b-5p, Col1a2, and CTGF in cardiac fibroblasts.^[10]^ Recently, circRNA profiles of IH were elucidated by microarray analysis,^[11]^ exhibiting the prospect of studying circRNAs in IHs.

In this study, we use RNA sequencing (RNA-Seq) and circRNA prediction to examine the expression profile of circRNA in 3 IH skin samples and a matched normal skin control. Subsequently, gene ontology and pathway analysis showed that compared to matched normal skin, many processes over-represented in IH are related to immune system processes, extracellular regio,n and molecular transducer activity. Our study may help expand the understanding of the roles of circRNAs in the mechanisms that underlie IH development and may provide new research directions.

## 2 Materials and methods

### 2.1 Ethics statement

This study was approved by the Medical Ethics Committee of the Affiliated Obstetrics and Gynecology Hospital of Nanjing Medical University (No. [2015]91). Children with IH attended our hospital for surgery. The IH samples and matched normal skins were collected from patients who underwent surgery with their parents’ written consent.

### 2.2 Tissue samples

Proliferating capillary IH and matched normal skin tissues were obtained from 3 different patients who were admitted to the Affiliated Obstetrics and Gynecology Hospital of Nanjing Medical University for IH removal. A diagnosis of proliferative IH was confirmed by routine pathological examination. The collected skin samples were immediately frozen in liquid nitrogen for the preparation of total RNA. Patient information is listed in Table 1.

**Table 1:**
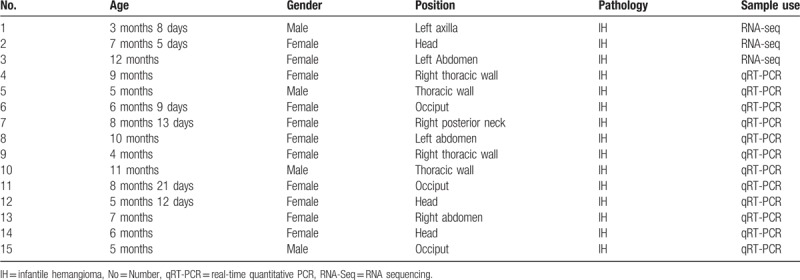
Demographic and clinical characteristics of IH patients (capillary hemangioma).

### 2.3 Total RNA isolation

Total RNA was extracted from biopsy samples using the Qiagen miRNeasy Mini Kit (Qiagen, Valencia, CA). RNA purity was checked using the NanoDrop 2000 (Thermo Fisher, MA), and RNA concentration was measured using the Qubit 3.0 Fluorometer (Life Technologies, CA). RNA integrity was assessed using the Agilent 2100 Bioanalyzer (Agilent Technologies, CA).

### 2.4 Library preparation, RNA-Sequencing

The transcriptome library for sequencing was generated using the VAHTS Total RNA-Seq (H/M/R) Library Prep Kit for Illumina (Vazyme Biotech Co., Ltd, Nanjing, China) following the manufacturer's recommendations. The details of the library construction were as follows: first, ribosomal RNA was removed by target-specific probes, RNase H and DNA polymerase I. Following purification, the RNA was fragmented into small pieces using divalent cations at elevated temperature. The cleaved RNA fragments were copied into the first strand cDNA using reverse transcriptase and random primers, followed by second-strand cDNA synthesis using DNA polymerase I, RNase H, and dNTPs (dUTP, dATP, dGTP, and dCTP). To these cDNA fragments were added a single ’A’ base, and the adapter was subsequently ligated. To select the appropriate cDNA fragment size for sequencing, the library fragments were selected with VAHTS DNA Clean Beads. The UDG enzyme was used to digest the second strand of cDNA. PCR amplification was performed, and the desired products were purified. After cluster generation, the libraries were sequenced on an Illumina Hiseq X10 platform, and 150-bp paired-end reads were generated.

### 2.5 Quality control

Raw reads in FASTQ format were first processed using in-house Perl scripts. Clean reads were obtained by removing reads with adapters, reads in which unknown bases were more than 5% and low-quality reads (ie, for which the percentage of low quality bases was over 50% in a read; we defined a low-quality base to be the base whose sequencing quality was at or below 10). At the same time, Q20, Q30, and GC content was calculated for the clean reads. All downstream analyses were based on the clean reads.

### 2.6 Mapping to the reference genome

The reference genome and gene model annotation files were downloaded directly from the UCSC (hg38). The reference genome index was built using Bowtie (v2.1.0),^[12]^ and paired-end clean reads were aligned to the reference genome using TopHat (v2.1.1).^[13]^

### 2.7 Raw data filtering

The raw reads were filtered by removing reads containing adapter, ploy-N and low-quality reads for subsequent analysis. The steps of sequencing data filtering were as follows: removing reads containing adapter; removing reads containing poly-N (ie, unrecognized bases), reads with a ratio greater than 5%; and removing low-quality reads (eg, for which the number of bases with Q≤10 is more than 50% of the entire read). All downstream analyses were based on clean data of high quality.

### 2.8 circRNA prediction

circRNA prediction was performed with circRNA_Finder (https://github.com/bioxfu/circRNAFinder).^[14]^ First, the clean reads were aligned to the reference genome using Bowtie2 (http://bowtie-bio.sourceforge.net/bowtie2/manual.shtml).^[12]^ Then, for unmapped reads, the junctions were selected using a back-splice algorithm and circRNAs were verified with the sub-module of circRNAFinder. Finally, circRNAs were annotated and abstracted with the circRNAAnno of circRNAFinder, which were considered the reference sequence for further analysis.

### 2.9 Differentially expressed circRNAs

The expression level of circRNAs was measured by “Transcripts Per Million” (TPM).^[15]^ Differentially expressed circRNAs were analyzed using the DESeq package based on the negative binomial distribution test. The thresholds of differentially expressed genes were FDR≤0.05 and │log2Fold change│ ≥1.

### 2.10 Validation in RNA-Seq data by quantitative reverse transcription-polymerase chain reaction (qRT-PCRs)

To confirm the RNA-Seq data, the expression of randomly selected circRNAs was tested in another 12 IH patients using qRT-PCRs with the SYBR green method on an Applied Biosystems ViiA Dx instrument (Life Technologies). Patient information is listed in Table 1. The sequences of specific PCR primer sets used for the qRT-PCR are listed in Table 2. The circRNA expression was normalized to the internal control gene, glyceraldehyde 3-phosphate dehydrogenase (GAPDH), using the 2^(-△△Ct)^ method.^[16]^

**Table 2:**
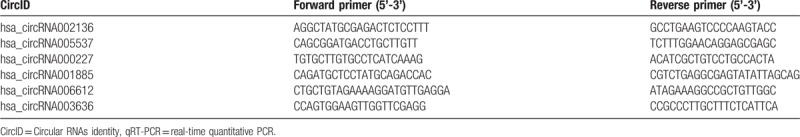
Details of primer pairs used in analysis of circRNAs expression by qRT-PCR.

### 2.11 GO enrichment and pathway analysis of origin genes of circRNAs

Using the statistical model, we performed gene ontology (GO) and Kyoto Encyclopedia of Genes and Genomes (KEGG) pathway analysis. Briefly, GO enrichment analysis of the origin genes of differentially expressed circRNAs was implemented with the GOseq R package, with gene length bias corrected. The resulting *P*-values were adjusted using Benjamini and Hochberg's approach for controlling the false discovery rate. GO terms with corrected *P* < .05 were considered significantly enriched among the differentially expressed genes. The top 10 GO terms are shown. We further used the KOBAS software program to test for the statistical enrichment of the origin genes of differentially expressed circRNAs among the KEGG pathways. The top 20 KEGG pathways are presented.

### 2.12 circRNA-microRNA interaction prediction

The circRNA-miRNA interaction was predicted with miRNA target prediction software^[17]^ (miRanda, http://www.microrna.org/). A Circos map of circRNA and miRNAs was drawn using a webserver (http://circos.ca/).

### 2.13 Statistical analysis

Data were analyzed using the SPSS 20.0 software package (SPSS, Chicago, IL) with an independent-sample *t* test between the 2 groups. All values were represented as the mean±standard deviation (SD) from at least 3 independent experiments. Statistical significance was defined as *P* < .05.

## 3 Results

### 3.1 CircRNA expression profile detected by RNA-Seq in IH compared to matched normal skin

**Figure 1:**
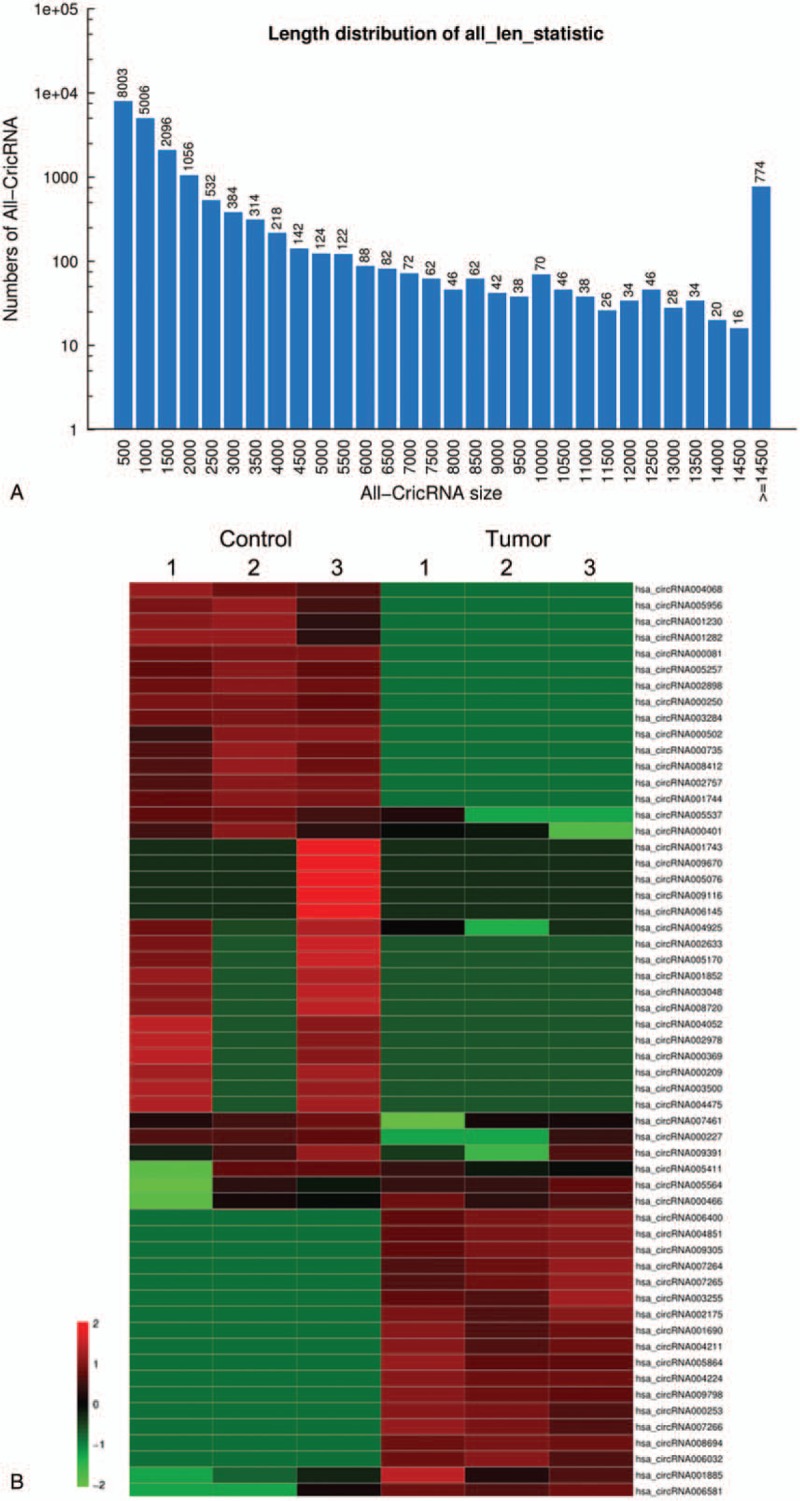
Expression profiles of circRNAs between infantile hemangioma and adjacent normal skin tissues. A, Length distribution of all circRNAs. B, Hierarchical clustering shows some of the 249 differentially expressed circRNAs among groups. Control, matched normal skin tissue; Tumor, infantile hemangioma skin tissue.

Skin samples were collected from 15 IH patients without previous treatment in the study. As shown in Table 1, the mean (SD) age of the IH patients was 7 months 6 days (2 months 15 days). To profile the differentially expressed circRNAs in IH, we performed RNA-Seq on 3 randomly selected IH samples and matched the normal skin. The length of circRNAs in all samples is shown in Figure 1 A. Most circRNAs were below 500 nucleotides. In all, 249 circRNAs were differentially expressed, including 124 upregulated and 125 downregulated circRNAs in IH compared to the matched normal skin control. Hierarchical clustering showed a list of the 249 differentially expressed circRNAs among the samples (Fig. 1 B). Table 3 shows part of the 249 differentially expressed circRNAs in the IH samples compared to the matched normal skin group (fold change ≥ 2, *P* ≤ .05).

**Table 3:**
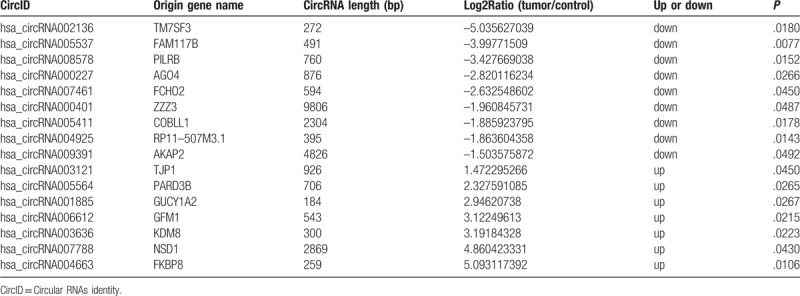
A list of differentially expressed circRNAs (fold change ≥ 2, *P* ≤ .05).

### 3.2 Real-time quantitative PCR validation

**Figure 2:**
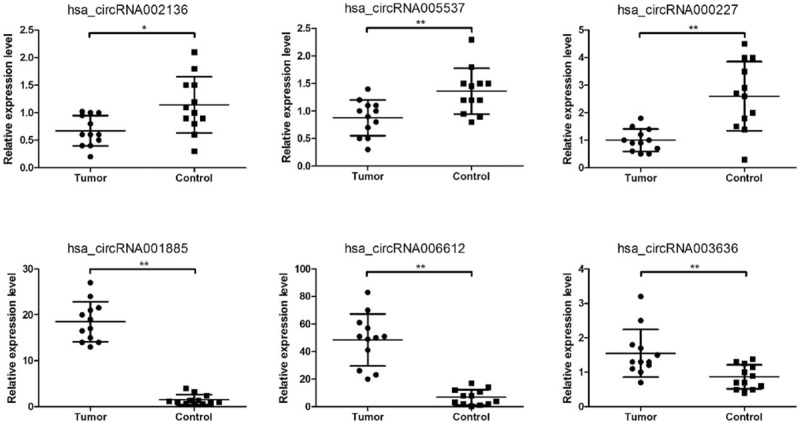
Differential expression of circRNAs between additional IH skin (n = 12) and matched normal skin tissues (n = 12). circRNA expression was validated by quantitative real-time PCR using 2^(-△△Ct)^ method. ∗*P* < .05, ∗∗*P* < .01.

To validate the RNA-Seq data, we randomly selected 6 differentially expressed circRNAs (bold in Table 3). Real-time quantitative PCR (qRT-PCR) analysis was performed on an additional 12 independent IH skin samples (Table 1). The results revealed that similar upregulation or downregulation was observed in both RNA-Seq and qRT-PCR samples for the 6 circRNAs (Fig. 2, bold in Table 3). Therefore, our RNA-Seq data were reliable and stable. Among the 6 circRNAs, hsa_circRNA001885, and hsa_circRNA006612 expression in IH was 12.33- and 7.13-fold higher, respectively, than in matched normal skin.

### 3.3 GO and KEGG pathway analysis of origin genes of circRNAs in IH compared to matched normal skin

**Figure 3:**
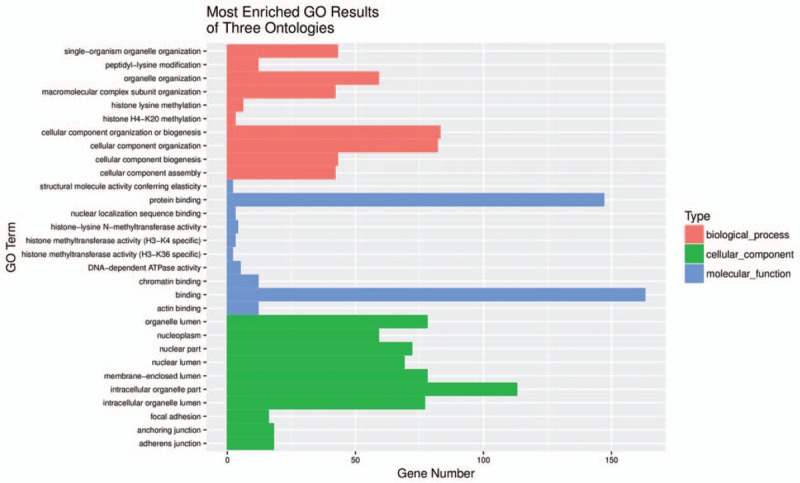
Gene ontology (GO) terms for origin genes of differentially expressed circRNAs between infantile hemangioma and adjacent normal skin tissues. Top 10 most enriched GO terms of three ontologies associated with the origin genes of differentially expressed circRNAs are listed. The horizontal axis represents the gene number. The term/GO on the vertical axis is drawn according to the first letter of the GO in descending order. Red bar represents biological process, blue bar displays molecular function, and green bar illustrates cellular component. GO = gene ontology.

**Figure 4:**
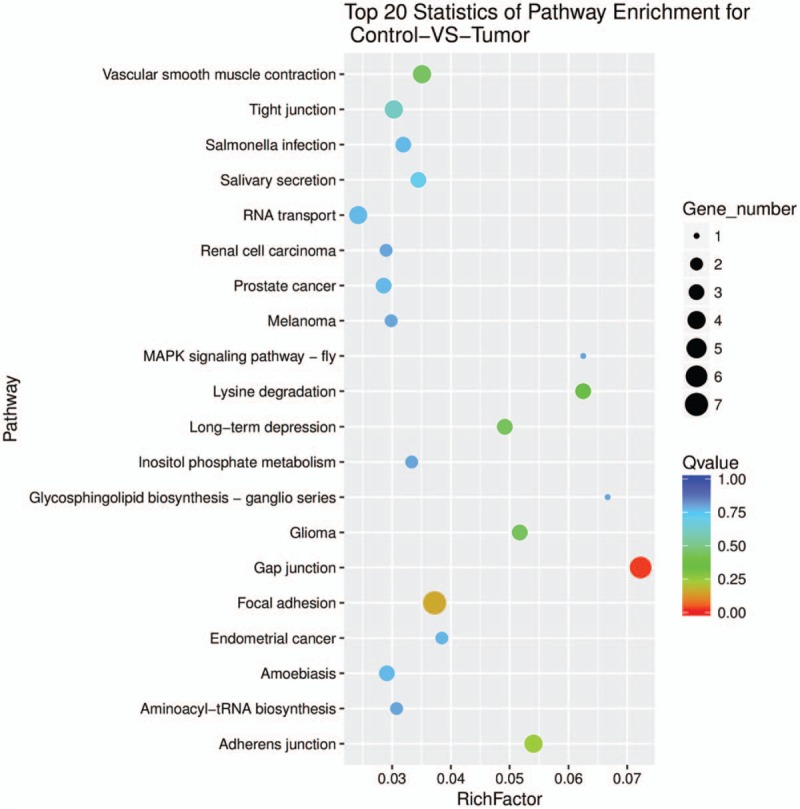
KEGG pathway analysis for origin genes of differentially expressed circRNAs between infantile hemangioma and adjacent normal skin tissues. The top 20 pathways are listed. The enrichment Q value or false discovery rate corrects the *P* value for multiple comparisons. *P* values are calculated using Fisher's exact test. The term/pathway on the vertical axis is drawn according to the first letter of the pathway in descending order. The horizontal axis represents the enrichment factor (ie, number of dysregulated genes in a pathway/the total number of dysregulated gene)/(number of genes in a pathway in database/the total number of genes in database). The top 20 enriched pathways are selected according to enrichment factor value. The selection standards were the number of genes in a pathway ≥ 4. The different colors from green to red represent the Q value (false discovery rate value). The different sizes of the round shapes represent the gene count number in a pathway. Control, matched normal skin tissue; Tumor, infantile hemangioma skin tissue.

Recent advances have revealed that circRNAs can regulate the expression of parental genes at the transcription level.^[18,19]^ Therefore, we analyze the origin genes of differentially expressed circRNAs through GO and KEGG pathway analysis. The GO results reveal that the origin genes of differentially expressed circRNAs are mostly involved in cellular component organization or biogenesis, binding, protein binding, and intracellular organelle parts (Fig. 3). The KEGG pathway analysis indicated that gap junction, focal adhesion, and adherens junction were implicated for the origin genes of differentially expressed circRNAs (Fig. 4).

### 3.4 Interaction between circRNA and miRNA

**Figure 5:**
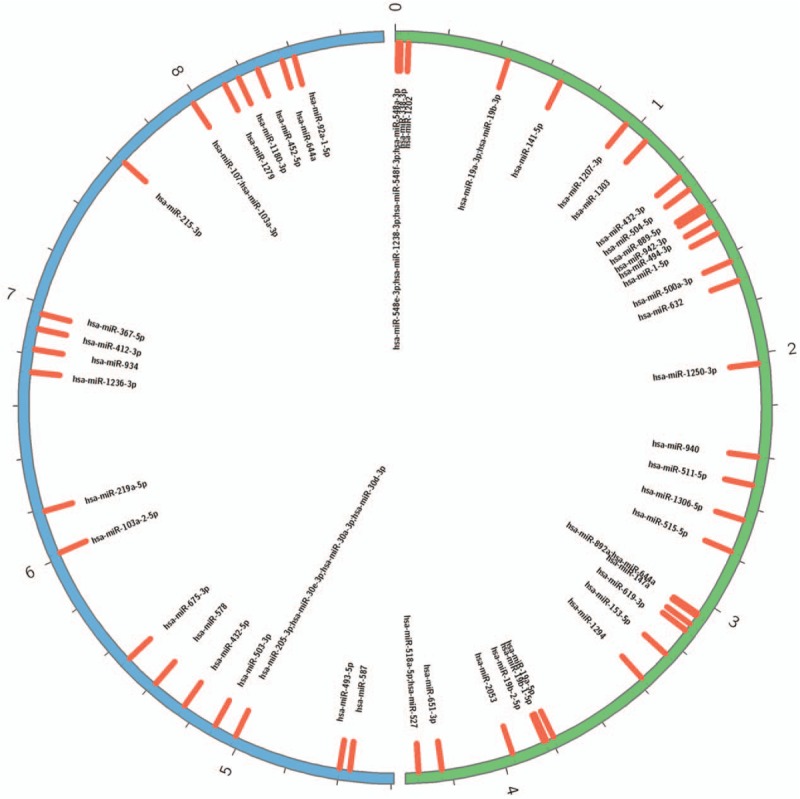
Circos map of potential interaction between hsa_circRNA000227 and 63 miRNAs.

Accumulated evidence indicates that circRNAs can function as miRNA sponges.^[20,21]^ The competitive endogenous RNAs (ceRNAs) contain shared miRNA response elements (MREs), such as circRNAs, messenger RNAs (mRNAs) and long noncoding RNAs (lncRNAs), and can compete for miRNA binding.^[22]^ Therefore, we use miRanda to screen the MREs in the 6 circRNAs validated. The results displayed several miRNAs associated with specific circRNAs (Table 4). A total of 63 miRNAs (the highest amount) could potentially bind with hsa_circRNA000227 (Fig. 5), 15 miRNAs could bind with hsa_circRNA001885 and 36 miRNAs could bind with hsa_circRNA006612.

**Table 4:**
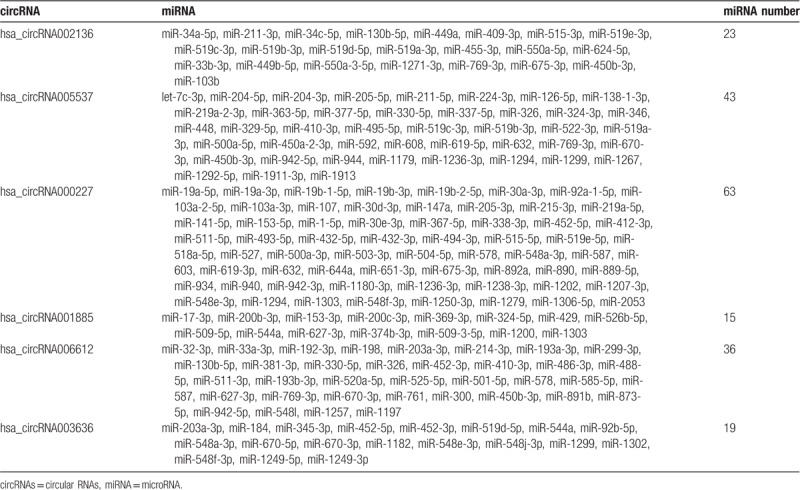
The interaction of circRNA and miRNAs was predicted using miRanda.

## 4 Discussion

As a vascular neoplasm, IH is one of the most common tumors diagnosed in young children.^[2]^ The pathogenesis of hemangioma has been widely studied, and several theories have been proposed, among which endothelial progenitor cell theory, Folkman Klagsbrun placental theory, angiogenesis theory, and hypoxia theory are the most accepted.^[23]^ To date, circRNA profiles by microarray analysis in the IHs have been reported,^[11]^ and 234 up- and 374 downregulated circRNAs were identified in IH by microarray.^[11]^ In this study, based on the RNA-Seq technique, we found that 249 circRNAs are dysregulated in IH including 124 upregulated and 125 downregulated circRNAs.

CircRNAs have been reported abundant, conserved and stable in cytoplasm,^[24]^ and originate from back splicing or exon skipping of linear RNA templates.^[8]^ Numerous articles describe thousands of circRNAs throughout RNA-Seq and back-splicing junction discovery to quantify circRNAs.^[24]^ A recent study proposed that the microarray is more efficient than RNA-Seq for circRNA profiling.^[25]^ Our circRNA profile in IHs was not completely consistent with the microarray data.^[11]^ According to the origin genes, we found that 47 origin genes of the 249 dysregulated circRNAs detected using RNA-Seq could be found in the circRNA microarray data (fold change ≥ 2, *P* < .5).

CircRNAs have recently emerged as novel star molecules that play crucial roles in the regulation of numerous biological or pathological processes.^[26,27]^ A recent study revealed that exon-intron circRNAs predominantly localize in the nucleus, interact with U1 snRNP and enhance the expression of their parental genes in cis.^[19]^ Our study shows that the origin genes of the 249 deregulated circRNAs are related to binding, protein binding, intracellular organelle part, gap junction, focal adhesion and adherens junction, indicating that circRNAs may function in these biological processes, molecular functions, and signaling pathways. Further elucidating the underlying mechanism of the function of circRNAs in IH would be helpful in revealing the biological aetiology and could provide useful information for IH evaluation and treatments.

For the origin genes of the validated 6 circRNAs (Table 3), we found that TM7SF3 and GUCY1A2 also could be found in the microarray data lists.^[11]^ Thus, hsa_circRNA002136 and hsa_circRNA001885 may be the most promising research objects. TM7SF3 can maintain protein homeostasis and promote cell survival through the attenuation of endoplasmic reticulum stress.^[28]^ Overexpression of TM7SF3 could inhibit caspase 3/7 activation.^[28]^ FAM117B has been a novel susceptibility locus identified with genome-wide significance in the European case-control populations.^[29]^ As an argonaute protein, Ago4 could bind small RNAs and mediate the cleavage of complementary target RNAs.^[30]^ GFM1 mutation could cause neonatal mitochondrial hepatoencephalopathy.^[31]^ JMJD5/KDM8 is important for genome stability in mammals.^[32]^ Therefore, specific circRNAs may affect IH in these ways. Further study is needed to reveal the function of specific circRNAs.

The circRNAs can sponge miRNAs by complementary base paring. A recent study reported that circHIPK3 could sponge 9 miRNAs with 18 potential binding sites.^[33]^ The circMTO1 could suppress hepatocellular carcinoma progression by acting as the sponge of oncogenic miR-9 to promote p21 expression, suggesting that circMTO1 is a potential target in HCC treatment.^[20]^ Our study finds that various miRNAs can bind specific circRNAs and mediate the roles of circRNAs in IH pathogenesis. Several studies reported that miR-130a, miR-382, miR-9, miR-939 and the let-7 family were involved in the pathogenesis of IH.^[34–36]^ Therefore, it is possible that hsa_circRNA005537 sponges to let-7c-3p and may have pivotal roles in the molecular mechanisms of IH development.

The functional interactions of circRNAs, miRNAs, and mRNAs could lead to a new explanation for the pathogenesis of diseases. Therefore, further elucidating the underlying mechanism of the function of miRNA, circRNAs, and mRNAs in IH would be helpful in revealing biological aetiology and potentially providing useful information for IH evaluation and treatments.

## Author contributions

Jun Li projected the experiment. Jingyun Li performed the sample preparation and wrote the manuscript. Qian Li, Ling Chen, and Yanli Gao performed the bioinformatics analysis. Jun Li edited the manuscript.

**Conceptualization:** Jun Li.

**Formal analysis:** Qian Li, Ling Chen, Yanli Gao.

**Funding acquisition:** Jun Li.

**Methodology:** Jingyun Li.

**Project administration:** Jingyun Li.

**Software:** Qian Li, Ling Chen, Yanli Gao.

**Supervision:** Jun Li.

**Writing – original draft:** Jingyun Li.

**Writing – review & editing:** Jun Li.

## References

[1] Munden A Butschek R Tom WL Prospective study of infantile haemangiomas: incidence, clinical characteristics and association with placental anomalies. Br J Dermatol 2014;170:907–13. 24641194 10.1111/bjd.12804 PMC4410180

[2] Itinteang T Withers AH Davis PF Biology of infantile hemangioma. Front Surg 2014;1:38. 25593962 10.3389/fsurg.2014.00038 PMC4286974

[3] Boscolo E Bischoff J Vasculogenesis in infantile hemangioma. Angiogenesis 2009;12:197–207. 19430954 10.1007/s10456-009-9148-2 PMC2810616

[4] Leaute-Labreze C Prey S Ezzedine K Infantile haemangioma: part I. Pathophysiology, epidemiology, clinical features, life cycle and associated structural abnormalities. J Eur Acad Dermatol Venereol 2011;25:1245–53. 21569112 10.1111/j.1468-3083.2011.04102.x

[5] Liu J Liu T Wang X Circles reshaping the RNA world: from waste to treasure. Mol Cancer 2017;16:58. 28279183 10.1186/s12943-017-0630-y PMC5345220

[6] Chen J Li Y Zheng Q Circular RNA profile identifies circPVT1 as a proliferative factor and prognostic marker in gastric cancer. Cancer Lett 2017;388:208–19. 27986464 10.1016/j.canlet.2016.12.006

[7] Tan WL Lim BT Anene-Nzelu CG A landscape of circular RNA expression in the human heart. Cardiovasc Res 2017;113:298–309. 28082450 10.1093/cvr/cvw250

[8] Yao T Chen Q Fu L Circular RNAs: Biogenesis, properties, roles, and their relationships with liver diseases. Hepatol Res 2017;47:497–504. 28185365 10.1111/hepr.12871

[9] Boeckel JN Jae N Heumuller AW Identification and characterization of hypoxia-regulated endothelial circular RNA. Circ Res 2015;117:884–90. 26377962 10.1161/CIRCRESAHA.115.306319

[10] Tang CM Zhang M Huang L CircRNA_000203 enhances the expression of fibrosis-associated genes by derepressing targets of miR-26b-5p, Col1a2 and CTGF, in cardiac fibroblasts. Sci Rep 2017;7:40342. 28079129 10.1038/srep40342 PMC5228128

[11] Fu C Lv R Xu G Circular RNA profile of infantile hemangioma by microarray analysis. Plos One 2017;12:e187581. 10.1371/journal.pone.0187581 PMC5667857 29095957

[12] Langmead B Salzberg SL Fast gapped-read alignment with Bowtie 2. Nat Methods 2012;9:357–9. 22388286 10.1038/nmeth.1923 PMC3322381

[13] Kim D Pertea G Trapnell C TopHat2: accurate alignment of transcriptomes in the presence of insertions, deletions and gene fusions. Genome Biol 2013;14:R36. 23618408 10.1186/gb-2013-14-4-r36 PMC4053844

[14] Hansen TB Veno MT Damgaard CK Comparison of circular RNA prediction tools. Nucleic Acids Res 2016;44:e58. 26657634 10.1093/nar/gkv1458 PMC4824091

[15] Zhou L Chen J Li Z Integrated profiling of microRNAs and mRNAs: microRNAs located on Xq27.3 associate with clear cell renal cell carcinoma. Plos One 2010;5:e15224. 21253009 10.1371/journal.pone.0015224 PMC3013074

[16] Li J Long W Li Q Distinct expression profiles of lncRNAs between regressive and mature scars. Cell Physiol Biochem 2015;35:663–75. 25613406 10.1159/000369727

[17] Enright AJ John B Gaul U MicroRNA targets in Drosophila. Genome Biol 2003;5:R1. 14709173 10.1186/gb-2003-5-1-r1 PMC395733

[18] Zhang Y Zhang XO Chen T Circular intronic long noncoding RNAs. Mol Cell 2013;51:792–806. 24035497 10.1016/j.molcel.2013.08.017

[19] Li Z Huang C Bao C Exon-intron circular RNAs regulate transcription in the nucleus. Nat Struct Mol Biol 2015;22:256–64. 25664725 10.1038/nsmb.2959

[20] Han D Li J Wang H Circular RNA MTO1 acts as the sponge of miR-9 to suppress hepatocellular carcinoma progression. Hepatology 2017;66:1151–64. 28520103 10.1002/hep.29270

[21] Wang K Gan TY Li N Circular RNA mediates cardiomyocyte death via miRNA-dependent upregulation of MTP18 expression. Cell Death Differ 2017;24:1111–20. 28498369 10.1038/cdd.2017.61 PMC5442477

[22] Qu S Yang X Li X Circular RNA: A new star of noncoding RNAs. Cancer Lett 2015;365:141–8. 26052092 10.1016/j.canlet.2015.06.003

[23] Abraham A Job AM Roga G Approach to infantile hemangiomas. Indian J Dermatol 2016;61:181–6. 27057018 10.4103/0019-5154.177755 PMC4817443

[24] Jakobi T Dieterich C Deep computational circular RNA analytics from RNA-seq data. Methods Mol Biol 2018;1724:9–25. 29322437 10.1007/978-1-4939-7562-4_2

[25] Li S Teng S Xu J Microarray is an efficient tool for circRNA profiling. Brief Bioinform 2018;[Epub ahead of print]. 10.1093/bib/bby006 29415187

[26] Memczak S Jens M Elefsinioti A Circular RNAs are a large class of animal RNAs with regulatory potency. Nature 2013;495:333–8. 23446348 10.1038/nature11928

[27] Hansen TB Jensen TI Clausen BH Natural RNA circles function as efficient microRNA sponges. Nature 2013;495:384–8. 23446346 10.1038/nature11993

[28] Isaac R Goldstein I Furth N TM7SF3, a novel p53-regulated homeostatic factor, attenuates cellular stress and the subsequent induction of the unfolded protein response. Cell Death Differ 2017;24:132–43. 27740623 10.1038/cdd.2016.108 PMC5260493

[29] Fischer A Ellinghaus D Nutsua M Identification of immune-relevant factors conferring sarcoidosis genetic risk. Am J Respir Crit Care Med 2015;192:727–36. 26051272 10.1164/rccm.201503-0418OC PMC4595678

[30] Hauptmann J Kater L Loffler P Generation of catalytic human Ago4 identifies structural elements important for RNA cleavage. RNA 2014;20:1532–8. 25114291 10.1261/rna.045203.114 PMC4174435

[31] Ravn K Schonewolf-Greulich B Hansen RM Neonatal mitochondrial hepatoencephalopathy caused by novel GFM1 mutations. Mol Genet Metab Rep 2015;3:5–10. 26937387 10.1016/j.ymgmr.2015.01.004 PMC4750589

[32] Amendola PG Zaghet N Ramalho JJ JMJD-5/KDM8 regulates H3K36me2 and is required for late steps of homologous recombination and genome integrity. Plos Genet 2017;13:e1006632. 28207814 10.1371/journal.pgen.1006632 PMC5336306

[33] Zheng Q Bao C Guo W Circular RNA profiling reveals an abundant circHIPK3 that regulates cell growth by sponging multiple miRNAs. Nat Commun 2016;7:11215. 27050392 10.1038/ncomms11215 PMC4823868

[34] Bertoni N Pereira LM Severino FE Integrative meta-analysis identifies microRNA-regulated networks in infantile hemangioma. Bmc Med Genet 2016;17:4. 26772808 10.1186/s12881-015-0262-2 PMC4715339

[35] Gao F Wang FG Liu RR Epigenetic silencing of miR-130a ameliorates hemangioma by targeting tissue factor pathway inhibitor 2 through FAK/PI3K/Rac1/mdm2 signaling. Int J Oncol 2017;50:1821–31. 28393235 10.3892/ijo.2017.3943

[36] Li D Li P Guo Z Downregulation of miR-382 by propranolol inhibits the progression of infantile hemangioma via the PTEN-mediated AKT/mTOR pathway. Int J Mol Med 2017;39:757–63. 28112362 10.3892/ijmm.2017.2863

